# Susceptibility to radiation adverse effects in veterans with Gulf War illness and healthy civilians

**DOI:** 10.1038/s41598-023-50083-7

**Published:** 2024-01-09

**Authors:** Beatrice Alexandra Golomb, Brinton Keith Berg, Jun Hee Han

**Affiliations:** Department of Medicine, UC San Diego School of Medicine, University of California, San Diego, 9500 Gilman Dr. #0995, La Jolla, CA 92093-0995 USA

**Keywords:** Medical research, Risk factors

## Abstract

We evaluated whether veterans with Gulf War illness (VGWI) report greater ionizing radiation adverse effects (RadAEs) than controls; whether radiation-sensitivity is tied to reported chemical-sensitivity; and whether environmental exposures are apparent risk factors for reported RadAEs (rRadAEs). 81 participants (41 VGWI, 40 controls) rated exposure to, and rRadAEs from, four radiation types. The relations of RadAE-propensity (defined as the ratio of rRadAEs to summed radiation exposures) to Gulf War illness (GWI) presence and severity, and to reported chemical-sensitivity were assessed. Ordinal logistic regression evaluated exposure prediction of RadAE-propensity in the full sample, in VGWI, and stratified by age and chemical-sensitivity. RadAE-propensity was increased in VGWI (vs. controls) and related to GWI severity (p < 0.01) and chemical-sensitivity (p < 0.01). Past carbon monoxide (CO) exposure emerged as a strong, robust predictor of RadAE-propensity on univariable and multivariable analyses (p < 0.001 on multivariable assessment, without and with adjustment for VGWI case status), retaining significance in age-stratified and chemical-sensitivity-stratified replication analyses. Thus, RadAE-propensity, a newly-described GWI-feature, relates to chemical-sensitivity, and is predicted by CO exposure—both features reported for nonionizing radiation sensitivity, consistent with shared mitochondrial/oxidative toxicity across radiation frequencies. Greater RadAE vulnerability fits an emerging picture of heightened drug/chemical susceptibility in VGWI.

## Introduction

There is need to understand susceptibility to effects of radiation^1–14^—radiosensitivity, radioresistance, and radiation toxicity, and factors tied to these^15–17^. This understanding may be important both for optimizing therapeutic benefit of radiation treatment (e.g., using radiosensitizers) and for protecting patients from medical misadventure. Moreover, the need to understand factors linked to radiation effect vulnerability extends beyond settings of therapeutic radiation use (e.g., occupational and military occupational settings). Radiation therapy for cancers have both capitalized intentionally on radiosensitizers^18^, and been plagued by complications arising from inadvertent harms of radiation particularly to radiosensitive individuals^17,19^. Findings from medical radiation have parallels with ultraviolet radiation toxicity (e.g., sunburn). In that setting, photosensitizing agents are known, and oxidative stress (OS) is implicated^20–23^. OS arises from and contributes to mitochondrial toxicity, which also serves as a known toxicity mechanism for radiation across the electromagnetic spectrum, extending also to nonionizing radiation^24–34^.

Chemical sensitivity (symptoms in response to levels of chemicals that are not a problem for most people) has been tied to heightened sensitivity to toxicity from nonionizing radiation^35,36^. This is consistent with the same shared mechanisms of toxicity involving OS and mitochondrial impairment, relevant again to radiation and to many drugs and chemicals, irrespective of their nominal specific modes of action^37–56^. Some exposures—like organophosphates—can lead to persistent mitochondrial toxicity^57–59^ and thereby ongoing elevated OS^60–62^, so could serve as instigators of persistent radiation sensitivity. To our knowledge, there has been no assessment of whether chemical sensitivity is tied to radiosensitivity (which is the term used to refer to “the relative susceptibility of cells, tissues, organs or organisms to the harmful effect of ionizing radiation”^63^, here used in reference to the organisms)—increased toxicity/adverse effects (AEs) with ionizing radiation exposure.

Some agents (such as fluoroquinolone antibiotics) have been tied to radiation sensitivity in many frequency bands—for (ionizing) medical radiation^64,65^, photosensitivity (ultraviolent radiation)^65–68^, and nonionizing radiation sensitivity^69^. (Fluoroquinolone mechanisms of action prominently involve OS and mitochondrial impairment^38^).

Gulf War illness (GWI) is an environmentally-triggered chronic multisymptom health condition that affects an estimated third of the ~ 700,000 US personnel deployed to the 1990–1991 Gulf theater—*attributable to* deployment^70^. It prominently involves fatigue, brain and muscle symptoms, with gastrointestinal, respiratory, dermatologic, pain, and autonomic symptoms also common. Dose–response and gene-environment data support causality for environmental exposures, particularly acetylcholinesterase inhibiting carbamates. GWI has been documented to involve mitochondrial impairment^71–73^. Veterans with GWI (VGWI) report increased intolerance/AEs to many drugs and environmental exposures^74^, including increased rates of self-reported chemical sensitivity^74–81^—particularly those veterans with pesticide exposure^81^. Organophosphates and carbamates were heavily used in the Gulf^82,83^, and mechanisms of toxicity again involve OS and mitochondrial compromise^84–89^. VGWI were heavily exposed to numerous environmental agents. Increased exposures and increased AEs in this group may afford improved opportunity/power to appraise exposure relations to radiation AEs (RadAEs).

Electromagnetic radiation comprises “transverse” waves (in which the direction of variation is perpendicular to the direction of travel, in contrast to longitudinal waves, such as sound waves). These travel at the speed of light, irrespective of the frequency of the radiation. Since all travel at the same speed (“C,” 2.99 × 10^8^ m/s), those with a longer wavelength (meters per cycle) must have a lower frequency (cycles per second) to achieve the same speed (meters per second or C). Higher frequencies of radiation have more energy, according to the equation E = hν, where “ν” (“nu”) is the frequency and “h,” the proportionality constant, is Planck’s constant. At high enough frequencies (energies), the energy suffices to dislodge an electron from an atom or molecule, a process termed “ionization.” Thus, higher frequencies—starting partway through the ultraviolet range and higher—are termed “ionizing” and lower frequencies termed “nonionizing.” Examples of nonionizing radiation include powerline radiation, radio waves, and microwaves (a subset of radio waves), such as are used in cell towers, cell phones and many communication devices. Examples of ionizing radiation include the higher frequencies of ultraviolet, as well as X-rays and gamma rays.

In fact, just because some radiation is ionizing does not mean that the ionization is responsible for its toxicity, and indeed most data on harms of (and protections against harms of) ionizing radiation relate not to the ionization but to oxidative stress—the type of free radical injury that antioxidants help to protect against—and oxidative stress is also shown for nonionizing radiation^25^ (both types also show interrelated biological mechanisms including mitochondrial injury, and membrane damage, for instance^24,90–101^). This paper is focused on ionizing radiation, but draws parallels to findings reported with nonionizing radiation. (While there is nominally debate about whether nonionizing radiation can cause biological and health effects, this is substantially a manufactured debate, with evidence showing powerful ties between results of studies and financial conflicts of interest^102,103^. The majority of publications that evaluate the relationship between anthropogenic nonionizing radiation and biological/health effects do show a relationship, despite the impact of financial conflicts of interest^104^).

In this effort, we wish to see if the increased reported propensity to experience AEs to drugs and environmental factors observed in VGWI extended to AEs of radiation. We seek to assess whether propensity to experiencing RadAEs relates to GWI severity or to chemical sensitivity. Finally, we wish to begin initial exploration of whether certain exposure factors might contribute to mediating increased radiation adverse effect propensity, if observed.

## Results

Table 1 shows participant characteristics. 93% of participants were male, 54% Caucasian. 41 GWI cases, and 40 controls matched to 40 of the cases were similar in age, sex, and ethnicity, by selection. Minor/nonsignificant differences were attributable to the additional unmatched case. VGWI were more likely to be married. (We’ve noted this in prior studies, and hypothesize that this is because VGWI are significantly compromised and it is primarily those with good social support that are able to add study participation to their already challenging lives). Cases were selected for meeting GWI symptom criteria, controls for not doing so. Thus, a total symptom score was much higher in affected veterans, as were a number of Kansas symptom domains that “qualified” toward GWI (out of a maximum of six). Self-reported chemical sensitivity was strongly greater in VGWI.

**Table 1:** Study participant characteristics (N = 81).

Characteristic	All N = 81		Case N = 41		Control N = 40		P*
	%		%		% %		Chi-2
Male	92.6		92.7		92.5		0.98
Caucasian^a^	54.3		53.7		55.0		0.90
Married	53.1		65.9		40.0		0.020
Chemical sensitivity	28.4		53.7		2.5		< 0.001

	Mean (SD)	Range	Mean (SD)	Range	Mean (SD)	Range	Chi-2
Age (years)	49.8 (7.5)	39–68	50.1 (7.56)	41–68	49.5 (7.52)	39–66	0.67

Gulf War illness symptoms and qualifying domains							
	Mean (SD)	Range	Mean (SD)	Range	Mean (SD)	Range	Chi-2
Total Kansas score	20.07 (22.5)	0–79	39.1 (16.1)	10–79	0.55 (0.96)	0–4	< 0.0001
# Qualifying Kansas domains	2.54 (2.52)	0–6	4.90 (1.00)	3–6	0.13 (0.33)	0–1	< 0.0001

Specific radiation exposures							
X-ray radiation	0.59 (0.49)	0–1	0.73 (0.43)	0–1	0.44 (0.50)	0–1	0.019
Radioactive chemicals	0.23 (0.35)	0–1	0.37 (0.39)	0–1	0.088 (0.25)	0–1	< 0.001
Radiation therapy	0.056 (0.21)	0–1	0.073 (0.24)	0–1	0.038 (0.18)	0–1	0.72
Other radiation	0.26 (0.40)	0–1	0.45 (0.44)	0–1	0.063 (0.202)	0–1	< 0.001

Aggregate radiation variables							
	Mean (SD)	Range	Mean (SD)	Range	Mean (SD)	Range	Chi-2
rRadAE	0.27 (0.58)	0–3.5	0.45 (0.75)	0–3.5	0.075 (0.21)	0–1	0.0021
Totrad	1.04 (0.97)	0.05–4	1.46 (1.06)	0.05–4	0.60 (0.61)	0.05, 2	< 0.0001
RadAE propensity	0.13 (0.23)	0–1	0.19 (0.24)	0–0.88	0.073 (0.21)	0, 1	0.0079

*GWI* Gulf War illness, *SD* standard deviation.

*P value is for case–control difference, and is based on Chi-2 test for binary variables, rank sum test for continuous variables.

^a^Representation of other ethnic groups included 20% black, 17% Hispanic, and 7% Asian, including some with mixed ethnicities in these values.

Totrad = total radiation score = sum of exposure across four radiation exposure types, each rated 0 = absent to 1 = present.

rRadAE = radiation symptom score = sum of adverse effects experienced across four radiation exposure types, each rated 0 = absent to 1 = present.

RadAE propensity = radiation adverse effect propensity = rRadAE/totrad.

As Table 1 also shows, reported radiation exposures were greater in veteran participants, with the radiation exposure score 2.4-fold higher in those with GWI. Radiation symptoms were sixfold greater in VGWI than healthy controls; and RadAE propensity score was about 2.5-fold greater. (RadAE reports were predominantly from x-ray radiation in both cases and controls).

Table 2A shows that RadAE propensity relates strongly to GWI severity (gauged here by the summed Kansas symptom score) in the total sample and cases separately, demonstrated by nonparametric test of trend and (unadjusted) ordinal logistic regression. GWI severity range in controls is rigidly restricted (by selection), precluding meaningful analysis in controls separately. Table 2B shows that RadAE propensity relates strongly to chemical sensitivity, in the total sample and in cases separately. Significance of the relationship is shown by rank sum test, and then by (univariable) ordinal logistic regression. As noted previously, only one control cited chemical sensitivity, precluding analysis in controls separately.

**Table 2:** Radiation adverse effect propensity^a^: clinical correlates.

(A) Relation of RadAE propensity to GWI severity^b^								
	Nptrend^c^		RadAE propensity prediction by GWI severity^d^					
	Z	P	OR (SE)	95% CI	P	Pseudo R^2^	Model P	
All (N = 81)	4.07	< 0.01	1.05 (0.016)	1.02, 1.08	0.001	0.096	0.0014	
Cases (N = 41)	3.25	< 0.01	1.08 (0.032)	1.02, 1.15	0.006	0.11	0.0060	

(B) Relation of RadAE propensity to chemical sensitivity^e^								
	RadAE propensity Mean (SD)		P Rank sum^c^	RadAE propensity prediction by chemical sensitivity^b^				
	Chemical sensitivity status			OR (SE)	95% CI	P	Pseudo R^2^	Model P
	Without	With						
All (N = 81)	0.075 (0.19)	0.27 (0.27)	0.0002	6.41 (3.46)	2.23, 18.4	0.001	0.071	0.0006
Cases (N = 41)	0.077 (0.16)	0.28 (0.27)	0.0074	6.22 (4.22)	1.65, 23.5	0.007	0.065	0.0070

*GWI* Gulf War illness, *OR* odds ratio, *SE* standard error, *CI* confidence interval, *P* probability.

^a^Radiation adverse effect propensity = summed radiation adverse effect score/summed radiation exposure score.

^b^Ordinal logit with robust standard errors. RadAE propensity as dependent variable. No adjustments.

^c^Nonparametric tests are performed due to skewed nature of variables. Analyses use the rank sum test for the binary variable (chemical sensitivity), and nonparametric test of trend (“nptrend”) for the multiple-value GWI severity variable. Significance digits provided (Stata) vary by tests: The nptrend test p-values were displayed as “0.00”.

^d^GWI severity: summed rating (0–3, absent, mild, moderate, severe) across the 28 symptoms of the Kansas GWI symptom criteria (1).

^e^Chemical sensitivity variable, binary (0 = absent, 1 = present).

Numerous exposures were assessed as candidate univariate predictors of RadAE propensity, overall and separately in cases and controls. The strongest univariable predictors are shown in Supplement Table 1. Highlighted exposures are those that were significant in the total sample, and in cases and controls separately (despite low power for this assessment in controls). Exposures were prioritized for display if they had a z-score of at least 2 in cases and a z-score of around 3 or greater in the total sample. These included carbon monoxide (CO) (strongest in both groups, by over a full z-score point, overall and in cases separately), some fuels/solvents, metals, pesticides/repellents, and radiation. Radiation exposures were predictors of RadAE propensity in cases and in the total sample, but not in controls. Two exposures are shown that were strong in controls (albeit based on small numbers) and significant in the total sample, but not in cases.

Table 3 shows results of a multivariable regression model predicting RadAE propensity with several predictor variables. CO, diesel fumes, and twinrx (hepatitis A + B) vaccine were selected based on assessing univariable predictors to RadAE propensity in a multivariable model. As in the univariable assessments for all participants and for cases, CO was the strongest predictor. Testing other variables from Supplement Table 1 by addition/substitution to this Primary Model served to support the Primary Model. Pesticide and metal variables appeared promising on univariable assessment but did not approach significance with adjustment for Primary Model variables, while CO and diesel fumes retained significance. Twinrx had a weaker univariable relationship, but performed better than other assessed variables in the multivariable model. As Table 3 shows, GWI case status was not a separately significant predictor of RadAE propensity after these exposures were considered. Assessing the model in cases separately, two of the three variables retained significance. The magnitude of the beta was similar or greater in cases for the third variable, but the sample was smaller and standard error for the variable larger. The model was assessed in the full sample with and without adjustment for case status; and in cases and controls separately. Table 3 also shows the multivariable model in split halves analysis stratified at the median age. CO retains significance in both age strata. The twinrx exposure was borderline significant in both age groups. Attempting reassessment of age-stratified analysis among cases, or with adjustment for cases, appeared to reproduce significance of CO in both age groups (p < 0.001), and produced significance for the twinrx vaccine in the full group adjusted for case status; however, convergence was not achieved in the younger age group for these models.

**Table 3:** Radiation adverse effect propensity^a^: multivariable prediction^b^.

Predictors	All participants, with adjustment for case status					
	Not adjusted for case status N = 81			Adjusted for case status N = 81		
	OR (SE)	95% CI	P	OR (SE)	95% CI	P
Carbon monoxide	27.9 (21.0)	6.41, 122	< 0.001	30.9 (23.7)	6.85, 139	< 0.001
Twinrx^c^	4.93 (3.13)	1.42, 17.1	0.012	5.59 (3.59)	1.59, 19.7	0.007
Diesel fumes	5.78 (4.66)	1.19, 28.0	0.030	9.87 (9.43)	1.52, 64.2	0.017
Case, GWI status				0.389 (0.332)	0.073, 2.07	0.27
Model performance	R^2^ = 0.27, P < 0.0001			R^2^ = 0.27, P < 0.0001		

Predictors	Stratified by case status					
	GWI cases N = 41			Controls N = 40		
	OR (SE)	95% CI	P	OR (SE)	95% CI	P
Carbon monoxide	82.8 (83.9)	11.4, 604	< 0.001	9.46 (10.3)	1.13, 79.5	0.039
Twinrx^c^	7.12 (5.82)	1.43, 35.3	0.016	4.00 (5.75)	0.238, 67.2	0.016
Diesel fumes	13.3 (22.8)	0.462, 384	0.13	6.57 (6.37)	0.981, 44.0	0.052
Model performance	R^2^ = 0.30, P < 0.0001			R^2^ = 0.19, p = 0.009		

Exposure	Age-stratified, not adjusted for GWI case status					
	Age < 48 N = 40			Age ≥ 48 N = 41		
	OR (SE)	95% CI	P	OR (SE)	95% CI	P
Carbon monoxide	30.5 (44.9)	1.71, 546	0.020	60.8 (61.3)	8.44, 438	< 0.001
Twinrx^c^	4.82 (3.89)	0.989, 23.5	0.052	9.18 (10.9)	0.905, 93.1	0.061
Diesel fume	20.6 (34.0)	0.801, 528	0.068	1.69 (1.38)	0.342, 8.36	0.52
Model performance	Pseudo R^2^ = 0.29, p = 0.0067			Pseudo R^2^ = 0.31, p = 0.0008		

*GWI* Gulf War illness, *OR* odds ratio, *SE* standard error, *CI* confidence interval, *P* probability.

^a^Radiation adverse effect propensity = summed radiation adverse effect score/summed radiation exposure score.

^b^Ordinal logit with robust standard errors.

^c^Three participants (two controls and one case) had missing twinrx values and were assigned a value of zero for twinrx, as this left findings substantially unchanged but allowed for use of the full dataset.

Table 4 shows the multivariable model in split halves analysis stratified at the median age. CO retains significance in both age strata. The twinrx exposure was borderline significant in both age groups. Attempting reassessment of age-stratified analysis among cases, or with adjustment for cases, appeared to reproduce significance of CO in both age groups (p < 0.001), and produced significance for the twinrx vaccine in the full group adjusted for case status; however, convergence was not achieved in the younger age group for these models.

**Table 4:** Radiation adverse effect propensity^a^: multivariable prediction stratified by chemical sensitivity^b^.

Exposure	No chemical sensitivity^c^ N = 58			Chemical sensitivity^c^ N = 23		
	OR (SE)	95% CI	P	OR (SE)	95% CI	P
Carbon monoxide	30.0 (32.5)	3.60, 250	0.002	43.6 (57.3)	3.32, 573	0.004
Twinrx vaccine	3.59 (2.91)	0.729, 17.6	0.12	6.42 (6.29)	0.943, 43.8	0.057
Diesel fume	2.60 (2.35)	0.440, 15.3	0.29	17.5 (31.2)	0.528, 578	0.11
*Model performance*	R^2^ = 0.24, Model p = 0.0080			R^2^ = 0.25, Model P = 0.0002		

*GWI* Gulf War illness, *OR* odds ratio, *SE* standard error, *CI* confidence interval, *P* probability.

^a^Radiation adverse effect propensity = summed radiation adverse effect score/summed radiation exposure score.

^b^Ordinal logit with robust standard errors. Combined GWI cases and controls.

^c^Chemical sensitivity variable, binary (0 = absent, 1 = present).

Table 4 shows the models stratified by chemical sensitivity. There were more participants without chemical sensitivity, but greater prevalence of radiation problems in those with chemical sensitivity. Significance for the CO relationship is observed in each group.

Supplement Table 2 shows that the relation of chemical sensitivity to RadAE propensity, and of GWI severity to RadAE propensity, each remained significant after adjustment for the model predictors, in the total sample (adjusted for case status), and in cases separately. The twinrx vaccine is significant in the model adjusted for chemical sensitivity but not GWI severity. Conversely, diesel fumes are significant in the model adjusted for GWI severity, but not the case model with adjustment for chemical sensitivity, consistent with collinearity between diesel fume exposure and chemical sensitivity. Strong significance for CO (p < 0.001) survives addition of these variables to the model. Case status emerges as a significant *negative* predictor of RadAE propensity in models adding adjustment for these variables.

## Discussion

Three principal findings emerge from this study. First, VGWI were more prone to reported AEs when exposed to radiation than were healthy controls, and this propensity was tied to the severity of GWI. Heightened vulnerability to radiation toxicity is thus a newly described feature of GWI. Second, while a tie between chemical sensitivity and vulnerability to symptoms from *nonionizing* radiation has previously been reported^35,36^, a novel finding here is that heightened vulnerability of persons with chemical sensitivity to radiation extends to *ionizing* radiation adverse effects, consistent with known shared mechanisms of OS and mitochondrial impairment, for toxicity of radiation both ionizing and nonionizing^24–34^, and toxicity of many drugs and chemicals^37–56,105^. Third, in the context that a hypothesized relation between CO exposure and vulnerability to nonionizing radiation has been previously reported^106^: here, CO exposure was the strongest and most robust predictor of *ionizing* RadAE propensity—robust to covariable adjustments and data stratifications.

Each of these findings triangulates with and receives support from other evidence. Increased vulnerability to reported RadAEs in GWI fits longstanding reports by VGWI that heightened vulnerability to drug and environmental toxicity is a feature of their illness—reports that now have substantiation in evidence^74,107^. Many drugs and chemicals, as well as both ionizing^15,17,18,108–128^ and nonionizing^24–31^ radiation, have toxicity mediated substantially *not* through the nominal specific action of the respective agent, but through OS and mitochondrial injury^37–56^. Exposures and conditions tied to mitochondrial injury (like CO^129–134^; GWI^71,73^; and conditions that overlap with GWI such as chronic fatigue syndrome^135^, which is also tied to mitochondrial impairment^135^) may lead to greater free radical production since mitochondria—especially when compromised^60–62^—are a leading source as well as a target of oxygen free radicals^60–62^, providing a lesser antioxidant buffer against new exposures. This may also thwart ability of new exposures to produce oxidative preconditioning—upregulation of antioxidant defenses—if such defenses are already maximally engaged or overwhelmed. It is likely at least in part for this reason that mitochondrial patients can fare poorly with many drugs^37,136,137^; indeed, “avoiding certain drugs is often more beneficial than application of established, apparently indicated drugs”^138^. Moreover, “mitochondrial cocktails,” used to mitigate clinical problems in affected patients, focus on antioxidation in addition to energy support^139,140^. Patients with “overlap” syndromes, like chemical sensitivity/fibromyalgia/irritable bowel syndrome, which are present with increased prevalence in those with GWI^75,78^, can also fare poorly on many drugs^141^. Findings shown here dictate the need to examine whether ionizing RadAE vulnerability may also be elevated in overlap conditions like irritable bowel syndrome.

Chemical sensitivity is among the overlap conditions tied to vulnerability to AEs of nonionizing radiation, and is particularly strongly related^35,142^. Nonionizing radiation can also produce AEs^104,143–148^ (emphasizing a vulnerable subset), and as noted above, toxicity of radiation (nonionizing *and* ionizing) as well as of many drugs and chemicals is mediated by OS and mitochondrial impairment, providing a foundation for strong crossover. Consistent with mediation by OS, gene polymorphisms and detoxifying activity related to less avid antioxidant defenses have been linked to both chemical sensitivity^149,150^ and “electrosensitivity”^151^. OS and impaired antioxidant defenses have also been tied to heightened toxicity of ionizing radiation^15,118^. Chemical sensitivity and electrosensitivity overlap with chronic multisymptom illnesses like GWI and chronic fatigue syndrome^69,152–154^—i.e., prevalence of each is elevated in those affected by the other—and mitochondrial impairment has been reported in these latter conditions^71,73,135,155^. This underscores the likelihood that mitochondrial compromise—produced also by CO^129,133,134,156,157^—is a predisposing factor^71,135^. To our knowledge, the data presented here are the first to empirically tie reported chemical sensitivity or any related “overlap condition” to increased vulnerability to AEs from ionizing radiation.

Potentially buttressing the striking relationship observed between CO exposure and RadAE vulnerability, one physician published a report of his own experience developing nonionizing radiation sensitivity (electrosensitivity) following sustained low-grade CO exposure^106^; and we are familiar with another case of severe electrosensitivity that followed a more acute CO exposure with persisting sequalae. In both instances, the individuals became intolerant to forms of radiation (nonionizing and in at least one, also ionizing) that they previously tolerated. In both, temporality was clear: the CO exposure preceded heightened vulnerability to radiation effects. Since CO can cause mitochondrial injury and enhances OS production^129,132–134^, and since mitochondrial injury can lead to ongoing OS^62,158^, perching the individual closer to (or beyond) the threshold at which further OS or exposures will surpass antioxidant defenses, a causal relationship to heightened radiosensitivity is strongly plausible. Indeed, mechanisms of injury with CO, including mitochondrial damage and OS^129,132–134^, are also present with known radiation sensitizing (including photosensitizing) agents, such as fluoroquinolone antibiotics^38,64,159–170^.

Data were drawn from a study with a different principal purpose^171,172^; however, the original study included broad inquiries precisely to permit analyses like this that stretch knowledge about GWI and open domains of inquiry. Veterans with Gulf War illness are affected by fatigue, so inquiries eliciting AE details were optional: While comment lines were provided for adverse effect descriptions, many participants did not make use of the opportunity to share these. Although the sample size of 81 is comparatively modest for evaluating relations of exposures to outcomes, a focus on a high-exposure/high-outcome VGWI group markedly enhances statistical power, and strong significance even on internal stratification underscores the adequacy of sample size/power for this purpose. This study has limitations, such as the cross-sectional character of exposure and AE elicitation. However, prospective studies are unlikely to occur eliciting exposure and adverse effect information beginning during high exposure military operations, when different mission objectives are the priority. Additionally, a conflict involving the high number and intensity of multiple exposures may (it is hoped) not recur—and these adverse features advantage power to examine exposure-outcome ascertainments, for a study such as this.

Veterans have more health problems and may have been exposed to more medical radiation, but this cannot account for the evidence, including the apparent relation of RadAE propensity to CO exposure, and its significant relation to multiple chemical sensitivity—which is known to be elevated in VGWI^75–78,80,81^. These factors comport with the hypothesis that mitochondrial impairment—implicated in GWI^71,73^, in radiation toxicity^24,173^, in CO toxicity^129,132–134^ and in chemical sensitivity^149^—are implicated here.

The present study, as above, is cross-sectional, and involves self-report: Self-report always carries the possibility of reporting or recall bias. However, self-reported exposures in VGWI have effectively been validated by potent evidence of gene-environment interactions^174,175^ based on self-reported exposures: Such potent interactions, with self-report conducted in absence of knowledge of genetic features, validate exposure self-report in VGWI on a statistical basis. Moreover, prior literature has reported good concordance between patient reports/attributions of AEs and AEs determined based on knowledgeable interviewers’ ascertainment^176,177^. Retrospective AE self-report has elsewhere mirrored findings from AE reporting system data, which relies predominantly on provider reporting^177,178^. We know of no discussion relating radiation to GWI, or evaluating radiation injury as a feature of GWI (beyond limited discussions of depleted uranium). Nor has there been attention to CO (or diesel fumes)—though exhaust from tent heaters was a known exposure^70^. These factors reduce the likelihood that reporting bias plays a major role in the observed relationships.

In principle, it cannot be excluded from this analysis alone that common vulnerabilities (such as impaired antioxidant defenses) underlie both development of radiation toxicity, and recognition/awareness that CO exposure has occurred. Added caution with radiation exposure in those with past CO exposure would still, however, be indicated under this hypothesis.

Not every potentially relevant exposure was assessed, and for those that were, not enough individuals may have had the exposure to allow relevance to RadAE propensity to be evaluated. Sample sizes placed limits on examination of multiple exposures concurrently in relation to outcomes. Some exposure categories in which univariable relationships did not survive multivariable adjustment in this study, such as metal and pesticide exposures, may still merit attention in future studies. VGWI have the conjoint advantage and disadvantage, for exposure-outcome assessments, that they had many exposures. The presence of exposures aid power/ability to see exposure relationships to outcomes. Although exposure multiplicity might also increase prospects that an exposure serves as a proxy for a correlated exposure (or exposures), or that the relevance of an exposure may be obscured through collinearity with another exposure, replication of key study findings in controls goes far towards mitigating this concern. Particularly for exposure relations, authority of findings will rest on replication and/or triangulation with other evidence.

While multiple analyses were performed, most served to affirm/validate key findings in different ways. For exposure predictors, these served to first identify candidate predictors, and then assessed the robustness of identified exposure predictors. This type of analysis multiplicity, rather than serving to increase chance as the basis of findings, instead aids in defending against chance as the likely explanation for key findings.

Findings, positioned in context of other evidence, support a shift from the construct of “ionizing radiation” to the more clinically relevant construct of “oxidizing radiation.” Findings underscore the existing recognition that differences in vulnerability to radiation effects exist^18–20,150^ and that heightened vulnerability is tied to certain clinical conditions (e.g., here chemical sensitivity, GWI) and may be fostered by certain exposures. This information is important for optimizing outcomes and minimizing iatrogenic complications with radiation use for diagnosis and therapy^1,2,64,65,179–195^ and is germane in considerations for environmental and occupational radiation exposures. OS may heighten radiosensitivity—which may be favorable for treating cancer, but is unfavorable for bystander toxicity to healthy tissue. Findings fit with evidence that antioxidants may protect from the tissue injury that can follow radiation exposure, including for diagnostic purposes.

Future studies should replicate and extend findings, expand evaluation of, and prospectively assess predictors (including other factors tied to OS and mitochondrial mechanisms), elicit details on RadAEs, and expand assessment of whether vulnerability factors for AEs are shared for ionizing and nonionizing radiation^35,196–198^. Animal studies should experimentally examine the impact of individual and multiple pre-exposures, to a range of candidate risk factors (particularly with potential for mitochondrial toxicity)—assessing whether these depress the intensity or duration of radiation required to produce evidence of radiation damage, considering both damage mechanisms—e.g., OS, mitochondrial alteration, membrane alteration, etc.—or clinical injury—e.g., radiation mucositis, etc. Such assessments should consider including animals with genetic variations adverse to OS defense (vulnerable hosts), as occurs in environmentally vulnerable people^18–20,150^.

This study, for the first time, documents that radiation adverse effect propensity is elevated in veterans with Gulf War illness, a highly chemically exposed group. For the first time, it ties propensity to ionizing radiation adverse effects, to propensity to chemical sensitivity—extending past reported connections between chemical sensitivity and nonionizing radiation toxicity. For the first time, it identifies carbon monoxide exposure as an apparent risk factor for ionizing radiation sensitivity, triangulating with past published evidence putatively connecting carbon monoxide to development of nonionizing radiation sensitivity. Although these findings are novel, they fit in a framework of evidence in which exposures that mediate toxicity by oxidative stress and mitochondrial impairment (as does radiation) may serve as risk factors for development of enhanced toxicity with radiation exposure. Findings fit with and have implications for protection from radiation injury and enhancing radiation therapy effectiveness. Increased caution may be prudent with use of diagnostic and therapeutic radiation, and occupational or incidental exposure to radiation, in VGWI, in persons with chemical sensitivity, and in those with past carbon monoxide exposure.

## Methods

### Ethics statement

Data acquisition was funded by the Department of Defense Congressionally Directed Medical Research Programs (GW093063). The study funders had no role in the study design, collection, analysis and interpretation of data, or the decision to publish. The study was approved by the UCSD Human Research Protections Program (protocol number # 100959), and all participants gave written informed consent. All methods were performed in accordance with relevant guidelines and regulations.

### Study design

This study uses data from the UCSD Gulf War illness study, a case–control study from which multiple findings have emerged^107,171,172,199,200^. Both case–control analyses and cross-sectional analyses (examining predictor-outcome relationships in the total sample and in cases and controls separately) are included. Given the limited funding allocated for GWI and the strain on veteran participants arising from study participation, it is strongly desirable to respect participants’ contribution by efforts to gain (from this participation) maximal information of relevance and importance to affected veterans—and potentially extending beyond them. Use of data from a previous study constrains the nature of the measures available; these nonetheless afford critical opportunities to open new domains of inquiry.

### Participants

Eighty-one participants comprised 41 VGWI, and 40 healthy controls matched 1:1 to 40 of the cases on sex, age (within 4 years), and ethnicity. An additional case completed the study; recruitment had continued until there were 40 matched pairs, and for this individual GWI case, a matched control had not at that time been identified. Although limited case–control comparisons are also included, most analyses here are cross-sectional and the additional case adds relevant information in this setting.

#### Cases

To qualify as a GWI case, veterans must have been deployed to the Persian Gulf theater of operations any time between August 1, 1990 and July 31, 1991. Veterans were additionally required to meet both Centers for Disease Control & Prevention (CDC) and Kansas symptom inclusion criteria for GWI^78,201^. CDC criteria require presence of symptoms for at least 6 months, arising during or after Gulf War participation, in at least two of the three domains of fatigue/sleep, mood-cognitive, and musculoskeletal^201^. The more discriminating and specific Kansas criteria require that symptoms have been present for at least six months, arising during or after Gulf deployment, in at least three of a suite of six categories comprising fatigue/sleep, pain, neurological, cognitive/mood, respiratory, gastrointestinal, and dermatologic^78^. For a symptom domain to qualify toward Kansas symptom criteria, the component symptoms must be at least moderate in severity (not mild) and/or there must be multiple symptoms within the category^78^.

#### Controls

Healthy non-veteran controls were drawn from the general population (using recruitment sources such as ResearchMatch and drawing on control participants from prior Gulf War illness studies). To qualify as a control, prospective participants were required to be non-veterans, meeting neither Kansas nor CDC symptom inclusion criteria for GWI, and additionally not meeting Kansas exclusion criteria (that is, they could not have other health conditions such as lupus or multiple sclerosis that could produce symptoms that could be confused for those of GWI, whether or not such symptoms were present). Controls were selected to match 1:1 to enrolled cases on sex, ethnicity, and age. A half-match for ethnicity was deemed to be qualifying, in recognition of the prevalence of mixed ethnicities. Age matching for matched pairs was within four years.

#### Preference for non-veteran healthy controls

Both veteran and non-veteran controls have limitations of different types. Veterans that are from the Gulf War era but were not deployed, were often selected for non-deployment for reasons that may compromise their validity as controls^202^. Moreover, many military will have had exposures that may bear shared mechanisms with exposures that triggered GWI problems. Examples include shared use of many vaccines, historical heavy use in the military of toxic pesticides, and potential for exposure to depleted uranium, burn pits etc., in other deployments. Therefore, other veterans may yield exposure histories and health histories intermediate between healthy non-veteran controls and affected Gulf War veterans. Deployed but healthy *Gulf War veterans* may be different in a distinct way, as these—if they bore full Gulf War exposures but nonetheless were healthy—may have distinct mechanisms/physiologies that provide (on a group basis) selective protection. For these reasons, despite distinct potential limitations, we have elected to use non-veteran healthy individuals as controls in our GWI studies^71,73,107,171,172,199,200^.

### Measurements

Surveys elicited information on demographics, adherence to/scoring on Kansas and CDC GWI criteria, chemical sensitivity, exposures, and adverse effects.

#### Exposures

Participants were asked whether they had experienced each of an extensive list of general exposures (non-Gulf specific). Exposure to each was designated by the participant as “no,” “unsure,” or “yes” (coded as 0, 0.5, and 1, respectively). For veterans, a further survey inquired about Gulf theater-specific exposures. However, to enable use of the full sample, only non-Gulf specific exposures (including in the Gulf) are considered here (necessary to have numbers required for split halves and other analyses). For each exposure, participants were asked whether an adverse effect had been experienced to the exposure and were offered the (optional) opportunity to provide details. For this study, only radiation exposures (below) and their reported adverse effects were the focus of the outcome measure. Other exposures were assessed as predictors of radiation adverse effect propensity.

#### Radiation exposures

Four radiation-related exposures were queried: radiation therapy, x-ray radiation, radioactive chemicals, and other radiation. Exposures were rated “no,” “unsure,” or “yes.” Those with an exposure were asked if they had experienced an AE to the exposure (“symptoms or conditions” attributed to the exposure) and were offered the optional opportunity to provide details. Examples of radiation AEs can include, for instance, dermatitis^6,13^, mucositis^6,9^, esophagitis^7^, enteritis^8^, proctitis^1,2^, as well as central nervous system sequelae (including from blood–brain barrier breach)^5^; and so-called “late effects” also adding further renal, pulmonary, pain, fibrosis, lymphedema, skin, and CNS effects among others that have been tied to ongoing oxidative stress triggered by the radiation^203,204^. Response options were “no,” “unsure,” or “yes,” coded as 0, 0.5, and 1, respectively (functionally, an ordinal variable). A score of rRadAEs summed responses on the radiation exposure AE queries. Radiation exposures were summed across the radiation categories (totrad), downgrading unsure responses to 0.25 (so that when summed, four unsure responses are required to achieve the same score as one certain one). To generate a proxy for RadAE propensity, the ratio of rRadAEs over totrad was calculated following the tradition of ratio measures^205^. Three participants cited no radiation exposure. The decision was made to assign these a RadAE propensity of zero.

#### Chemical sensitivity

The study assessed self-rated chemical sensitivity via the chemical sensitivity question from the Kansas GWI questionnaire (recommended for GWI evaluation by both the Department of Defense^206^ and the Institute of Medicine/National Academy of Medicine^207^), as well as via our single-item UCSD GWI chemical sensitivity self-rating, analyzed as a binary assessment (0 if absent, 1 if present). The Kansas criteria question states: “Having physical or mental symptoms after breathing in certain smells or chemicals.” The timeframe for the Kansas query is the prior 6 months, with a 4-point Likert scale as absent, mild, moderate, severe rated 0, 1, 2, 3, respectively. The UCSD self-rating states: “Chemical sensitivity (e.g., unusual sensitivity to smells).” The timeframe is 2 weeks. The Kansas and UCSD measures show convergent validation against one another: r = 0.57, p = 0.0001. The single-item UCSD chemical sensitivity measure was further validated by affirming a previously reported relationship of chemical sensitivity to the polymorphism of the main mitochondrial antioxidant—SOD2, in which alanine rather than valine is present at codon 16^208^. This relationship was previously reported in a Japanese sample of paper pulp workers, that employed the QEESI chemical sensitivity ascertainment instrument (Japanese language version)^149^. Finally, the UCSD binary measure showed superior convergent validity relative to the Kansas instrument in correlating to actual chemical adverse effect propensity: UCSD r = 0.44, p = 0.004; Kansas r = 0.31, p = 0.047.

*GWI severity* was gauged by the proxy of summed Kansas criteria symptom scores^78^. Twenty-eight symptoms were each scored from 0 to 3 (as absent, mild, moderate, severe), and the ratings were summed.

### Analyses

Descriptive statistics were used to depict participant characteristics, Kansas symptom ratings, totrad, rRadAE, RadAE propensity, GWI severity, and chemical sensitivity for all participants, and in cases and controls separately. Rank sum and chi-squared tests compared characteristics of cases to those of controls for continuous and categorical variables, respectively.

Relation of RadAE propensity to GWI severity score was assessed using nonparametric test of trend. Nonparametric tests were used due to skewed distributions. The relationship was assessed in the total sample and in cases separately. Controls were selected for very low and tightly restricted GWI severity scores, precluding separate analysis of these relationships in controls.

Relation of RadAE propensity to self-rated chemical sensitivity was assessed using the rank sum test, in all participants and in cases separately. Only one control cited chemical sensitivity, precluding separate analysis of this relationship in controls. RadAE propensity took on 10 ordinally progressing values, and each of the two clinical relationships were reassessed using (unadjusted) ordinal logit. All regressions used robust (heteroskedasticity-independent) standard errors.

Univariable exposure relations to RadAE propensity were evaluated in the total sample, and in cases and controls separately, using nonparametric test of trend. Separate assessment in cases was important to ensure that any relations did not derive simply from higher exposure in cases, and concurrent higher RadAE propensity in cases. Predictors with z-scores exceeding 2 in cases, and around or above 3 in the total sample were assessed for inclusion in a multivariable model.

A set of three candidate predictors that retained significance in a multivariable model were then evaluated in all participants (with and without adjustment for case status), and in cases separately. (Controls did not support separate multivariable assessment). The model was appraised in split halves analysis, stratified at the median age. It was also assessed stratified by presence/absence of reported chemical sensitivity. Impact of addition of chemical sensitivity and of GWI severity to the models was evaluated.

For one of the exposure variables used in the multivariable model (hepatitis A + B vaccine i.e., twinrx), three participants did not provide a response. Missing values were coded as zero (no exposure, the most common reported exposure status for the variable), to allow use of the full data set. No other exposure variables among those in multivariable analyses, and no other variables used in analyses, had missing values.

Multiple comparison adjustment was not performed in this analysis. This is a novel analysis opening an area of inquiry, a setting in which Type II error is the bigger concern. Additionally, multiple hypothesis adjustment is based on the presumption that chance is the first order explanation for findings, which is commonly not the case in real world data of this type^209^. For case–control comparisons, 41 and 40 participants nominally provide 80% power with 2-sided alpha of 0.05 to detect an effect that is at least 0.63 standard deviations. De facto, each analysis involves different power considerations due to different fractions of participants with an exposure or outcome. Our emphasis is on findings that are robust.

Analyses used Stata^®^ versions 8.0 and 13.0 (College Station, Texas). Two-sided p < 0.05 designated statistical significance.

### Supplementary Information

Supplementary Information.

## Data Availability

Adequately deidentified data used and analyses shown in the current study are available from the corresponding author on reasonable request. Requests for data should be made to Dr. Golomb (bgolomb@ucsd.edu).

## References

[1] Wang L, Zhang ZZ, Tu XH, Zou ZD, Liu JH, Wang Y (2009). Safety and efficacy of Qingre Buyi Decoction in the treatment of acute radiation proctitis: A prospective, randomized and controlled trial. Chin. J. Integr. Med..

[2] Sahakitrungruang C, Thum-Umnuaysuk S, Patiwongpaisarn A, Atittharnsakul P, Rojanasakul A (2011). A novel treatment for haemorrhagic radiation proctitis using colonic irrigation and oral antibiotic administration. Colorectal Dis..

[3] Autio E, Saikkonen J (1963). Cobalt, copper and iron in anemia associated with radiotherapy of tumors. Preliminary communication. Acta Radiol. Ther. Phys. Biol..

[4] Georgieva B, Gekova K, Arsov T (1982). Effect of various cytostatics and radiotherapy on serum iron and copper levels in lymphoma patients. Vutr Boles.

[5] van Vulpen M, Kal HB, Taphoorn MJ, El-Sharouni SY (2002). Changes in blood-brain barrier permeability induced by radiotherapy: Implications for timing of chemotherapy? (Review). Oncol. Rep..

[6] Burdelya LG, Gleiberman AS, Toshkov I, Aygun-Sunar S, Bapardekar M, Manderscheid-Kern P, Bellnier D, Krivokrysenko VI, Feinstein E, Gudkov AV (2012). Toll-like receptor 5 agonist protects mice from dermatitis and oral mucositis caused by local radiation: Implications for head-and-neck cancer radiotherapy. Int. J. Radiat. Oncol. Biol. Phys..

[7] Topkan E, Yavuz MN, Onal C, Yavuz AA (2009). Prevention of acute radiation-induced esophagitis with glutamine in non-small cell lung cancer patients treated with radiotherapy: Evaluation of clinical and dosimetric parameters. Lung Cancer.

[8] Vidal-Casariego A, Calleja-Fernandez A, Cano-Rodriguez I, Cordido F, Ballesteros-Pomar MD (2015). Effects of oral glutamine during abdominal radiotherapy on chronic radiation enteritis: A randomized controlled trial. Nutrition.

[9] Tsujimoto T, Yamamoto Y, Wasa M, Takenaka Y, Nakahara S, Takagi T, Tsugane M, Hayashi N, Maeda K, Inohara H (2015). l-glutamine decreases the severity of mucositis induced by chemoradiotherapy in patients with locally advanced head and neck cancer: A double-blind, randomized, placebo-controlled trial. Oncol. Rep..

[10] Jones CU, Hunt D, McGowan DG, Amin MB, Chetner MP, Bruner DW, Leibenhaut MH, Husain SM, Rotman M, Souhami L (2011). Radiotherapy and short-term androgen deprivation for localized prostate cancer. N. Engl. J. Med..

[11] Schellart NA, Reits D, van der Kleij AJ, Stalpers LJ (2011). Hyperbaric oxygen treatment improved neurophysiologic performance in brain tumor patients after neurosurgery and radiotherapy: A preliminary report. Cancer.

[12] Wedlake L, Thomas K, McGough C, Andreyev HJ (2008). Small bowel bacterial overgrowth and lactose intolerance during radical pelvic radiotherapy: An observational study. Eur. J. Cancer.

[13] Schreck U, Paulsen F, Bamberg M, Budach W (2002). Intraindividual comparison of two different skin care conceptions in patients undergoing radiotherapy of the head-and-neck region. Creme or powder?. Strahlenther. Onkol..

[14] Talbot AR, Barnes MR (1988). Radiotherapy for the treatment of pulmonary complications of paraquat poisoning. Hum. Toxicol..

[15] Tulard A, Hoffschir F, de Boisferon FH, Luccioni C, Bravard A (2003). Persistent oxidative stress after ionizing radiation is involved in inherited radiosensitivity. Free Radic. Biol. Med..

[16] Cleaver JE (1989). How many human genetic disorders affect cellular radiosensitivity?. Cancer Cells.

[17] Mangoni M, Bisanzi S, Carozzi F, Sani C, Biti G, Livi L, Barletta E, Costantini AS, Gorini G (2011). Association between genetic polymorphisms in the XRCC1, XRCC3, XPD, GSTM1, GSTT1, MSH2, MLH1, MSH3, and MGMT genes and radiosensitivity in breast cancer patients. Int. J. Radiat. Oncol. Biol. Phys..

[18] Lee S, Lim MJ, Kim MH, Yu CH, Yun YS, Ahn J, Song JY (2012). An effective strategy for increasing the radiosensitivity of human lung cancer cells by blocking Nrf2-dependent antioxidant responses. Free Radic. Biol. Med..

[19] Andreassen CN (2010). Searching for genetic determinants of normal tissue radiosensitivity—Are we on the right track?. Radiother. Oncol..

[20] Dalle Carbonare M, Pathak MA (1992). Skin photosensitizing agents and the role of reactive oxygen species in photoaging. J. Photochem. Photobiol. B.

[21] Bickers DR, Athar M (2006). Oxidative stress in the pathogenesis of skin disease. J. Investig. Dermatol..

[22] Xiao YF, Chen WC, Chen JX, Lu G, Tian S, Cui X, Zhang Z, Chen H, Wan Y, Li S (2022). Amplifying free radical generation of AIE photosensitizer with small singlet-triplet splitting for hypoxia-overcoming photodynamic therapy. ACS Appl. Mater. Interfaces.

[23] González S, Pathak MA (1996). Inhibition of ultraviolet-induced formation of reactive oxygen species, lipid peroxidation, erythema and skin photosensitization by polypodium leucotomos. Photodermatol. Photoimmunol. Photomed..

[24] Golomb BA (2018). Diplomats' mystery illness and pulsed radiofrequency/microwave radiation. Neural Comput..

[25] Yakymenko I, Tsybulin O, Sidorik E, Henshel D, Kyrylenko O, Kyrylenko S (2015). Oxidative mechanisms of biological activity of low-intensity radiofrequency radiation. Electromagn. Biol. Med..

[26] Aydin B, Akar A (2011). Effects of a 900-MHz electromagnetic field on oxidative stress parameters in rat lymphoid organs, polymorphonuclear leukocytes and plasma. Arch. Med. Res..

[27] Bahreymi Toossi, M. H. *et al.* Exposure to mobile phone (900–1800 MHz) during pregnancy: Tissue oxidative stress after childbirth. *J. Matern. Fetal Neonatal Med.* 1–6 (2017) (**Epub ahead of print**). 10.1080/14767058.2017.1315657 28434276

[28] Bilgici B, Akar A, Avci B, Tuncel OK (2013). Effect of 900 MHz radiofrequency radiation on oxidative stress in rat brain and serum. Electromagn. Biol. Med..

[29] Deshmukh PS, Banerjee BD, Abegaonkar MP, Megha K, Ahmed RS, Tripathi AK, Mediratta PK (2013). Effect of low level microwave radiation exposure on cognitive function and oxidative stress in rats. Indian J. Biochem. Biophys..

[30] Devrim E, Erguder IB, Kilicoglu B, Yaykasli E, Cetin R, Durak I (2008). Effects of electromagnetic radiation use on oxidant/antioxidant status and DNA turn-over enzyme activities in erythrocytes and heart, kidney, liver, and ovary tissues from rats: Possible protective role of vitamin C. Toxicol. Mech. Methods.

[31] Esmekaya MA, Ozer C, Seyhan N (2011). 900 MHz pulse-modulated radiofrequency radiation induces oxidative stress on heart, lung, testis and liver tissues. Gen. Physiol. Biophys..

[32] Kostyuk SV, Proskurnina EV, Konkova MS, Abramova MS, Kalianov AA, Ershova ES, Izhevskaya VL, Kutsev SI, Veiko NN (2021). Effect of low-dose ionizing radiation on the expression of mitochondria-related genes in human mesenchymal stem cells. Int. J. Mol. Sci..

[33] Rai Y, Anita, Kumari N, Singh S, Kalra N, Soni R, Bhatt AN (2021). Mild mitochondrial uncoupling protects from ionizing radiation induced cell death by attenuating oxidative stress and mitochondrial damage. Biochim. Biophys. Acta Bioenergy.

[34] Miranda S, Correia M, Dias AG, Pestana A, Soares P, Nunes J, Lima J, Máximo V, Boaventura P (2020). Evaluation of the role of mitochondria in the non-targeted effects of ionizing radiation using cybrid cellular models. Sci. Rep..

[35] Belpomme D, Campagnac C, Irigaray P (2015). Reliable disease biomarkers characterizing and identifying electrohypersensitivity and multiple chemical sensitivity as two etiopathogenic aspects of a unique pathological disorder. Rev. Environ. Health.

[36] Hillert L, Berglind N, Arnetz BB, Bellander T (2002). Prevalence of self-reported hypersensitivity to electric or magnetic fields in a population-based questionnaire survey. Scand. J. Work Environ. Health.

[37] Golomb BA, Evans MA (2008). Statin adverse effects: A review of the literature and evidence for a mitochondrial mechanism. Am. J. Cardiovasc. Drugs.

[38] Golomb BA, Koslik HJ, Redd AJ (2015). Fluoroquinolone-induced serious, persistent, multisymptom adverse effects. BMJ Case Rep..

[39] Amin A, Hamza AA (2005). Oxidative stress mediates drug-induced hepatotoxicity in rats: A possible role of DNA fragmentation. Toxicology.

[40] Das GC, Bacsi A, Shrivastav M, Hazra TK, Boldogh I (2006). Enhanced gamma-glutamylcysteine synthetase activity decreases drug-induced oxidative stress levels and cytotoxicity. Mol. Carcinog..

[41] Denicola A, Radi R (2005). Peroxynitrite and drug-dependent toxicity. Toxicology.

[42] Fosslien E (1998). Adverse effects of nonsteroidal anti-inflammatory drugs on the gastrointestinal system. Ann. Clin. Lab. Sci..

[43] McMillian M, Nie A, Parker JB, Leone A, Kemmerer M, Bryant S, Herlich J, Yieh L, Bittner A, Liu X (2005). Drug-induced oxidative stress in rat liver from a toxicogenomics perspective. Toxicol. Appl. Pharmacol..

[44] Shuhendler AJ, Pu K, Cui L, Uetrecht JP, Rao J (2014). Real-time imaging of oxidative and nitrosative stress in the liver of live animals for drug-toxicity testing. Nat. Biotechnol..

[45] Tafazoli S, Spehar DD, O'Brien PJ (2005). Oxidative stress mediated idiosyncratic drug toxicity. Drug Metab. Rev..

[46] Verma P, Bhattacharya SN, Banerjee BD, Khanna N (2012). Oxidative stress and leukocyte migration inhibition response in cutaneous adverse drug reactions. Indian J. Dermatol. Venereol. Leprol..

[47] Joshi G, Sultana R, Tangpong J, Cole MP, St Clair DK, Vore M, Estus S, Butterfield DA (2005). Free radical mediated oxidative stress and toxic side effects in brain induced by the anti cancer drug adriamycin: Insight into chemobrain. Free Radic. Res..

[48] Kovacic P, Cooksy AL (2005). Unifying mechanism for toxicity and addiction by abused drugs: Electron transfer and reactive oxygen species. Med. Hypotheses.

[49] Varga ZV, Ferdinandy P, Liaudet L, Pacher P (2015). Drug-induced mitochondrial dysfunction and cardiotoxicity. Am. J. Physiol. Heart Circ. Physiol..

[50] Bastianon C, Zanoni R, Miolo G, Caffieri S, Reddi E (2005). Mitochondria and plasma membrane as targets of UVA-induced toxicity of neuroleptic drugs fluphenazine, perphenazine and thioridazine. Int. J. Biochem. Cell Biol..

[51] Boelsterli UA (2007). Lim: Mitochondrial abnormalities—A link to idiosyncratic drug hepatotoxicity?. Toxicol. Appl. Pharmacol..

[52] de Mendoza C, Sanchez-Conde M, Ribera E, Domingo P, Soriano V (2004). Could mitochondrial DNA quantitation be a surrogate marker for drug mitochondrial toxicity?. AIDS Rev..

[53] Finsterer J, Zarrouk-Mahjoub S (2015). Mitochondrial toxicity of cardiac drugs and its relevance to mitochondrial disorders. Expert Opin. Drug Metab. Toxicol..

[54] Foli A, Benvenuto F, Piccinini G, Bareggi A, Cossarizza A, Lisziewicz J, Lori F (2001). Direct analysis of mitochondrial toxicity of antiretroviral drugs. AIDS.

[55] Swartz MN (1995). Mitochondrial toxicity—New adverse drug effects. N. Engl. J. Med..

[56] Wallace KB, Starkov AA (2000). Mitochondrial targets of drug toxicity. Annu. Rev. Pharmacol. Toxicol..

[57] Middlemore-Risher ML, Adam BL, Lambert NA, Terry AV (2011). Effects of chlorpyrifos and chlorpyrifos-oxon on the dynamics and movement of mitochondria in rat cortical neurons. J. Pharmacol. Exp. Ther..

[58] Feng Y, Cui X, Yin J, Shao B (2022). Chlorinated organophosphorus flame retardants-induced mitochondrial abnormalities and the correlation with progesterone production in mLTC-1 cells. Food Chem. Toxicol..

[59] Piel S, Janowska JI, Ward JL, McManus MJ, Jose JS, Starr J, Sheldon M, Clayman CL, Elmér E, Hansson MJ (2022). Succinate prodrugs in combination with atropine and pralidoxime protect cerebral mitochondrial function in a rodent model of acute organophosphate poisoning. Sci. Rep..

[60] Genova ML, Pich MM, Bernacchia A, Bianchi C, Biondi A, Bovina C, Falasca AI, Formiggini G, Castelli GP, Lenaz G (2004). The mitochondrial production of reactive oxygen species in relation to aging and pathology. Ann. N. Y. Acad. Sci..

[61] Wei YH (1998). Oxidative stress and mitochondrial DNA mutations in human aging. Proc. Soc. Exp. Biol. Med..

[62] Lee HC, Wei YH (1997). Role of mitochondria in human aging. J. Biomed. Sci..

[63] Radiosensitivity. https://en.wikipedia.org/wiki/Radiosensitivity.

[64] Jain S, Agarwal J, Laskar S, Gupta T, Shrivastava S (2008). Radiation recall dermatitis with gatifloxacin: A review of literature. J. Med. Imaging Radiat. Oncol..

[65] Dawson GA, Brown SI, Tellefsen L (2009). A drug-related phototoxic reaction and its possible relationship to a radiation-induced skin reaction. Oncologist.

[66] Snyder RD, Cooper CS (1999). Photogenotoxicity of fluoroquinolones in Chinese hamster V79 cells: Dependency on active topoisomerase II. Photochem. Photobiol..

[67] Trisciuoglio D, Krasnowska E, Maggi A, Pozzi R, Parasassi T, Sapora O (2002). Phototoxic effect of fluoroquinolones on two human cell lines. Toxicol. In Vitro.

[68] Agrawal N, Ray RS, Farooq M, Pant AB, Hans RK (2007). Photosensitizing potential of ciprofloxacin at ambient level of UV radiation. Photochem. Photobiol..

[69] Golomb BA: Electrosensitivity: A 'current' and future problem. *Meeting: Cell Phones and Wireless Technologies—Should Safety Guidelines Be Strengthened to Protect Adults, Children and Vulnerable Populations?* (Commonwealth Club, 2015).

[70] Binns JH (2008). Gulf War Illness and the Health of Gulf War Veterans. Scientific Findings and Recommendations.

[71] Koslik HJ, Hamilton G, Golomb BA (2014). Mitochondrial dysfunction in Gulf War illness revealed by 31phosphorus magnetic resonance spectroscopy: A case-control study. PLoS ONE.

[72] Shetty GA, Hattiangady B, Upadhya D, Bates A, Attaluri S, Shuai B, Kodali M, Shetty AK (2017). Chronic oxidative stress, mitochondrial dysfunction, Nrf2 activation and inflammation in the hippocampus accompany heightened systemic inflammation and oxidative stress in an animal model of Gulf War illness. Front. Mol. Neurosci..

[73] Golomb BA, Sanchez Baez R, Schilling JM, Dhanani M, Fannon MJ, Berg BK, Miller BJ, Taub PR, Patel HH (2023). Mitochondrial impairment but not peripheral inflammation predicts greater Gulf War illness severity. Sci. Rep..

[74] Golomb BA, Han JH (2023). Adverse effect propensity: A new feature of Gulf War illness predicted by environmental exposures. iScience.

[75] Gray GC, Reed RJ, Kaiser KS, Smith TC, Gastanaga VM (2002). Self-reported symptoms and medical conditions among 11,868 Gulf War-era veterans: The Seabee Health Study. Am. J. Epidemiol..

[76] Thomas HV, Stimpson NJ, Weightman AL, Dunstan F, Lewis G (2006). Systematic review of multi-symptom conditions in Gulf War veterans. Psychol. Med..

[77] Unwin C, Blatchley N, Coker W, Ferry S, Hotopf M, Hull L, Ismail K, Palmer I, David A, Wessely S (1999). Health of UK servicemen who served in Persian Gulf War. Lancet.

[78] Steele L (2000). Prevalence and patterns of Gulf War illness in Kansas veterans: Association of symptoms with characteristics of person, place, and time of military service. Am. J. Epidemiol..

[79] Cherry N, Creed F, Silman A, Dunn G, Baxter D, Smedley J, Taylor S, Macfarlane GJ (2001). Health and exposures of United Kingdom Gulf war veterans. Part I: The pattern and extent of ill health. Occup. Environ. Med..

[80] Bell IR, Warg-Damiani L, Baldwin CM, Walsh ME, Schwartz GE (1998). Self-reported chemical sensitivity and wartime chemical exposures in Gulf War veterans with and without decreased global health ratings. Mil. Med..

[81] Reid S, Hotopf M, Hull L, Ismail K, Unwin C, Wessely S (2001). Multiple chemical sensitivity and chronic fatigue syndrome in British Gulf War veterans. Am. J. Epidemiol..

[82] Fricker, R. D. *et al.**Pesticide Use During the Gulf War: A Survey of Gulf War Veterans* (RAND, MR-1018/12-OSD, 2000).

[83] Cecchine, G., Golomb, B. A., Hilborne, L. H., Spektor, D. M. & Anthony, R. A. *A Review of the Scientific Literature as it Pertains to Gulf War Illnesses, Vol 8: Pesticides*. (RAND, 2000).

[84] Le Y, Shen H, Yang Z, Lu D, Wang C (2021). Comprehensive analysis of organophosphorus flame retardant-induced mitochondrial abnormalities: Potential role in lipid accumulation. Environ. Pollut..

[85] Farkhondeh T, Mehrpour O, Forouzanfar F, Roshanravan B, Samarghandian S (2020). Oxidative stress and mitochondrial dysfunction in organophosphate pesticide-induced neurotoxicity and its amelioration: A review. Environ. Sci. Pollut. Res. Int..

[86] Leung MCK, Meyer JN (2019). Mitochondria as a target of organophosphate and carbamate pesticides: Revisiting common mechanisms of action with new approach methodologies. Reprod. Toxicol..

[87] Peña-Llopis S, Ferrando MD, Peña JB (2003). Fish tolerance to organophosphate-induced oxidative stress is dependent on the glutathione metabolism and enhanced by N-acetylcysteine. Aquat. Toxicol..

[88] Peña-Llopis S, Ferrando MD, Peña JB (2003). Increased recovery of brain acetylcholinesterase activity in dichlorvos-intoxicated European eels *Anguilla anguilla* by bath treatment with N-acetylcysteine. Dis. Aquat. Organ..

[89] Milatovic D, Gupta RC, Aschner M (2006). Anticholinesterase toxicity and oxidative stress. ScientificWorldJournal.

[90] Schwarz W, Fox JM (1979). Effects of monochromatic X-radiation on the membrane of nodes of Ranvier under voltage and current clamp conditions. Experientia.

[91] Bhosle SM, Pandey BN, Huilgol NG, Mishra KP (2002). Membrane oxidative damage and apoptosis in cervical carcinoma cells of patients after radiation therapy. Methods Cell Sci..

[92] Shonai T, Adachi M, Sakata K, Takekawa M, Endo T, Imai K, Hareyama M (2002). MEK/ERK pathway protects ionizing radiation-induced loss of mitochondrial membrane potential and cell death in lymphocytic leukemia cells. Cell Death Differ..

[93] Benderitter M, Vincent-Genod L, Pouget JP, Voisin P (2003). The cell membrane as a biosensor of oxidative stress induced by radiation exposure: A multiparameter investigation. Radiat. Res..

[94] Kvam E, Dahle J (2005). The pheomelanin precursor 5-S-cysteinyldopa protects melanocytes from membrane damage induced by ultraviolet A radiation. Cancer Lett..

[95] Falzone N, Huyser C, Fourie F, Toivo T, Leszczynski D, Franken D (2008). In vitro effect of pulsed 900 MHz GSM radiation on mitochondrial membrane potential and motility of human spermatozoa. Bioelectromagnetics.

[96] Кhyzhnyak SV, Bezdrobna LK, Stepanova LI, Morozova VS, Voitsitskiy VM (2014). Oxidative phosphorylation in mitochondria of small-intestinal enterocytes at chronic and single exposure to low power ionizing radiation. Probl. Radiac. Med. Radiobiol..

[97] Ibrahim AA, Karam HM, Shaaban EA, Safar MM, El-Yamany MF (2019). MitoQ ameliorates testicular damage induced by gamma irradiation in rats: Modulation of mitochondrial apoptosis and steroidogenesis. Life Sci..

[98] Yoshida T, Goto S, Kawakatsu M, Urata Y, Li TS (2012). Mitochondrial dysfunction, a probable cause of persistent oxidative stress after exposure to ionizing radiation. Free Radic. Res..

[99] Burlaka A, Selyuk M, Gafurov M, Lukin S, Potaskalova V, Sidorik E (2014). Changes in mitochondrial functioning with electromagnetic radiation of ultra high frequency as revealed by electron paramagnetic resonance methods. Int. J. Radiat. Biol..

[100] Xie Y, Jiang HH, Gong QF, Zhang GB, Yu JH, Yu ZP (2004). Effect of microwave irradiation on neurocyte mitochondrial ultrastructure and mtTFA mRNA expression in rats cerebral cortex and hippocampus. Zhonghua Lao Dong Wei Sheng Zhi Ye Bing Za Zhi.

[101] Ramundo-Orlando A (2009). Effects of millimeter waves radiation on cell membrane—A brief review. J. Infrared Milli Terahz Waves.

[102] Huss A, Egger M, Hug K, Huwiler-Müntener K, Röösli M (2007). Source of funding and results of studies of health effects of mobile phone use: Systematic review of experimental studies. Environ. Health Perspect..

[103] Lai, H. Does mobile phone use affect your health? Keynote address. In *Symposium on Effects of Electromagnetic Fields on Public Health and Environment*, (Yildiz Teknik Universitesi, 2013).

[104] Bandara P, Carpenter DO (2018). Planetary electromagnetic pollution: It is time to assess its impact. Lancet Planet Health.

[105] Golomb, B. A. Oxidative stress and mitochondrial injury in chronic multisymptom conditions: From Gulf War illness to autism spectrum disorder. *Nature Precedings *citeseerxistpsuedu/viewdoc/download?10116656426&rep=rep1&type=pdf (2012).

[106] Eberle, S. What's the diagnosis, doctor? *Bulletin* **22**(6) (2016).

[107] Golomb BA, Nguyen E, Dinkeloo E (2020). Radiation exposure predicts reported vaccine adverse effects in veterans with Gulf War illness. Int. J. Environ. Res. Public Health.

[108] Simone G, Tamba M, Quintiliani M (1983). Role of glutathione in affecting the radiosensitivity of molecular and cellular systems. Radiat. Environ. Biophys..

[109] Malaise EP (1983). Reduced oxygen enhancement of the radiosensitivity of glutathione-deficient fibroblasts. Radiat. Res..

[110] Vos O, van der Schans GP, Roos-Verheij WS (1986). Reduction of intracellular glutathione content and radiosensitivity. Int. J. Radiat. Biol. Relat. Stud. Phys. Chem. Med..

[111] Edgren MR (1987). Nuclear glutathione and oxygen enhancement of radiosensitivity. Int. J. Radiat. Biol. Relat. Stud. Phys. Chem. Med..

[112] Hodgkiss RJ, Stratford MR, Watfa RR (1989). The effect of alpha-tocopherol and alpha-tocopheryl quinone on the radiosensitivity of thiol-depleted mammalian cells. Int. J. Radiat. Oncol. Biol. Phys..

[113] Vallis KA (1991). Glutathione deficiency and radiosensitivity in AIDS patients. Lancet.

[114] Yi X, Ding L, Jin Y, Ni C, Wang W (1994). The toxic effects, GSH depletion and radiosensitivity by BSO on retinoblastoma. Int. J. Radiat. Oncol. Biol. Phys..

[115] Orta T, Eady JJ, Peacock JH, Steel GG (1995). Glutathione manipulation and the radiosensitivity of human tumour and fibroblast cell lines. Int. J. Radiat. Biol..

[116] Mansur DB, Kataoka Y, Grdina DJ, Diamond AM (2001). Radiosensitivity of mammalian cell lines engineered to overexpress cytosolic glutathione peroxidase. Radiat. Res..

[117] Bravard A, Ageron-Blanc A, Alvarez S, Drane P, Le Rhun Y, Paris F, Luccioni C, May E (2002). Correlation between antioxidant status, tumorigenicity and radiosensitivity in sister rat cell lines. Carcinogenesis.

[118] Kato K, Takahashi K, Monzen S, Yamamoto H, Maruyama A, Itoh K, Kashiwakura I (2010). Relationship between radiosensitivity and Nrf2 target gene expression in human hematopoietic stem cells. Radiat. Res..

[119] Bardak Y, Ozerturk Y, Ozguner F, Durmus M, Delibas N (2000). Effect of melatonin against oxidative stress in ultraviolet-B exposed rat lens. Curr. Eye Res..

[120] Bruskov VI, Karp OE, Garmash SA, Shtarkman IN, Chernikov AV, Gudkov SV (2012). Prolongation of oxidative stress by long-lived reactive protein species induced by X-ray radiation and their genotoxic action. Free Radic. Res..

[121] Demir U, Demir T, Ilhan N (2005). The protective effect of alpha-lipoic acid against oxidative damage in rabbit conjunctiva and cornea exposed to ultraviolet radiation. Ophthalmologica.

[122] El-Missiry MA, Fayed TA, El-Sawy MR, El-Sayed AA (2007). Ameliorative effect of melatonin against gamma-irradiation-induced oxidative stress and tissue injury. Ecotoxicol. Environ. Saf..

[123] Goswami S, Haldar C (2014). Melatonin improves ultraviolet B-induced oxidative damage and inflammatory conditions in cutaneous tissue of a diurnal Indian palm squirrel Funambulus pennanti. Br. J. Dermatol..

[124] Jang SS, Kim HG, Lee JS, Han JM, Park HJ, Huh GJ, Son CG (2013). Melatonin reduces X-ray radiation-induced lung injury in mice by modulating oxidative stress and cytokine expression. Int. J. Radiat. Biol..

[125] Kim BC, Shon BS, Ryoo YW, Kim SP, Lee KS (2001). Melatonin reduces X-ray irradiation-induced oxidative damages in cultured human skin fibroblasts. J. Dermatol. Sci..

[126] Mishra KP (2004). Cell membrane oxidative damage induced by gamma-radiation and apoptotic sensitivity. J. Environ. Pathol. Toxicol. Oncol..

[127] Saada HN, Rezk RG, Eltahawy NA (2010). Lycopene protects the structure of the small intestine against gamma-radiation-induced oxidative stress. Phytother. Res..

[128] Sharma S, Haldar C (2006). Melatonin prevents X-ray irradiation induced oxidative damagein peripheral blood and spleen of the seasonally breeding rodent, *Funambulus pennanti* during reproductively active phase. Int. J. Radiat. Biol..

[129] Alonso JR, Cardellach F, Lopez S, Casademont J, Miro O (2003). Carbon monoxide specifically inhibits cytochrome c oxidase of human mitochondrial respiratory chain. Pharmacol. Toxicol..

[130] Hattori H, Sugawara N, Nakamura K, Furuno J (1990). The metabolic effect of carbon monoxide on the heart. Mol. Cell Biochem..

[131] Hattori H, Suzuki Y, Fujimiya T, Yamamoto K, Ueda M (1986). Acute effects of carbon monoxide and cyanide on hepatic mitochondrial function. Z Rechtsmed.

[132] Miro O, Casademont J, Barrientos A, Urbano-Marquez A, Cardellach F (1998). Mitochondrial cytochrome c oxidase inhibition during acute carbon monoxide poisoning. Pharmacol. Toxicol..

[133] Piantadosi CA, Carraway MS, Suliman HB (2006). Carbon monoxide, oxidative stress, and mitochondrial permeability pore transition. Free Radic. Biol. Med..

[134] Zhang J, Piantadosi CA (1992). Mitochondrial oxidative stress after carbon monoxide hypoxia in the rat brain. J. Clin. Investig..

[135] Myhill S, Booth NE, McLaren-Howard J (2009). Chronic fatigue syndrome and mitochondrial dysfunction. Int. J. Clin. Exp. Med..

[136] Ahn MS, Sims KB, Frazier JA (2005). Risperidone-induced psychosis and depression in a child with a mitochondrial disorder. J. Child Adolesc. Psychopharmacol..

[137] Finsterer J, Segall L (2010). Drugs interfering with mitochondrial disorders. Drug Chem. Toxicol..

[138] Finsterer J (2006). Central nervous system manifestations of mitochondrial disorders. Acta Neurol. Scand..

[139] Parikh S, Saneto R, Falk MJ, Anselm I, Cohen BH, Haas R, Medicine Society TM (2009). A modern approach to the treatment of mitochondrial disease. Curr. Treat. Options Neurol..

[140] Tarnopolsky MA (2008). The mitochondrial cocktail: Rationale for combined nutraceutical therapy in mitochondrial cytopathies. Adv. Drug Deliv. Rev..

[141] Poitras P, Gougeon A, Binn M, Bouin M (2008). Extra digestive manifestations of irritable bowel syndrome: Intolerance to drugs?. Dig. Dis. Sci..

[142] Bergqvist, U. *et al.* (eds.) Possible health implications of subjective symptoms and electromagnetic fields. A report prepared by a European group of experts for the European Commission, DG V. (European Commission Directorate General V. Employment, Industrial Relations and Social Affairs, National Institute for Working Life, 1997).

[143] Russell CL (2018). 5 G wireless telecommunications expansion: Public health and environmental implications. J. Environ. Res..

[144] Li Y, Héroux P (2014). Extra-low-frequency magnetic fields alter cancer cells through metabolic restriction. Electromagn. Biol. Med..

[145] Zelt RG, Daniel RK, Ballard PA, Brissette Y, Heroux P (1988). High-voltage electrical injury: Chronic wound evolution. Plast. Reconstr. Surg..

[146] Davis DL, Kesari S, Soskolne CL, Miller AB, Stein Y (2013). Swedish review strengthens grounds for concluding that radiation from cellular and cordless phones is a probable human carcinogen. Pathophysiology.

[147] Hardell L, Sage C (2008). Biological effects from electromagnetic field exposure and public exposure standards. Biomed. Pharmacother..

[148] Choi YJ, Moskowitz JM, Myung SK, Lee YR, Hong YC (2020). Cellular phone use and risk of tumors: Systematic review and meta-analysis. Int. J. Environ. Res. Public Health.

[149] Cui X, Lu X, Hiura M, Oda M, Miyazaki W, Katoh T (2013). Evaluation of genetic polymorphisms in patients with multiple chemical sensitivity. PLoS ONE.

[150] De Luca C, Raskovic D, Pacifico V, Thai JC, Korkina L (2011). The search for reliable biomarkers of disease in multiple chemical sensitivity and other environmental intolerances. Int. J. Environ. Res. Public Health.

[151] De Luca C, Chung Sheun Thai J, Raskovic D, Cesareo E, Caccamo D, Trukhanov A, Korkina L (2014). Metabolic and genetic screening of electromagnetic hypersensitive subjects as a feasible tool for diagnostics and intervention. Mediators Inflamm..

[152] Aaron LA, Buchwald D (2001). A review of the evidence for overlap among unexplained clinical conditions. Ann. Intern. Med..

[153] Brown MM, Jason LA (2007). Functioning in individuals with chronic fatigue syndrome: Increased impairment with co-occurring multiple chemical sensitivity and fibromyalgia. Dyn. Med..

[154] Kipen HM, Fiedler N (2002). Environmental factors in medically unexplained symptoms and related syndromes: The evidence and the challenge. Environ. Health Perspect..

[155] Booth NE, Myhill S, McLaren-Howard J (2012). Mitochondrial dysfunction and the pathophysiology of Myalgic Encephalomyelitis/Chronic Fatigue Syndrome (ME/CFS). Int. J. Clin. Exp. Med..

[156] Jang DH, Piel S, Greenwood JC, Kelly M, Mazandi VM, Ranganathan A, Lin Y, Starr J, Hallowell T, Shofer FS (2021). Alterations in cerebral and cardiac mitochondrial function in a porcine model of acute carbon monoxide poisoning. Clin. Toxicol..

[157] Jang DH, Khatri UG, Shortal BP, Kelly M, Hardy K, Lambert DS, Eckmann DM (2018). Alterations in mitochondrial respiration and reactive oxygen species in patients poisoned with carbon monoxide treated with hyperbaric oxygen. Intensive Care Med. Exp..

[158] Sastre J, Pallardo FV, Vina J (2003). The role of mitochondrial oxidative stress in aging. Free Radic. Biol. Med..

[159] Ghaly H, Jorns A, Rustenbeck I (2014). Effect of fluoroquinolones on mitochondrial function in pancreatic beta cells. Eur. J. Pharm. Sci..

[160] Hsiao CJ, Younis H, Boelsterli UA (2010). Trovafloxacin, a fluoroquinolone antibiotic with hepatotoxic potential, causes mitochondrial peroxynitrite stress in a mouse model of underlying mitochondrial dysfunction. Chem. Biol. Interact..

[161] Lowes DA, Wallace C, Murphy MP, Webster NR, Galley HF (2009). The mitochondria targeted antioxidant MitoQ protects against fluoroquinolone-induced oxidative stress and mitochondrial membrane damage in human Achilles tendon cells. Free Radic. Res..

[162] Pouzaud F, Dutot M, Martin C, Debray M, Warnet JM, Rat P (2006). Age-dependent effects on redox status, oxidative stress, mitochondrial activity and toxicity induced by fluoroquinolones on primary cultures of rabbit tendon cells. Comp. Biochem. Physiol. C Toxicol. Pharmacol..

[163] Pouzaud F, Bernard-Beaubois K, Thevenin M, Warnet JM, Hayem G, Rat P (2004). In vitro discrimination of fluoroquinolones toxicity on tendon cells: Involvement of oxidative stress. J. Pharmacol. Exp. Ther..

[164] Pouzaud F, Christen MO, Warnet JM, Rat P (2004). Anethole dithiolethione: An antioxidant agent against tenotoxicity induced by fluoroquinolones. Pathol. Biol..

[165] Kaleagasioglu F, Olcay E (2012). Fluoroquinolone-induced tendinopathy: Etiology and preventive measures. Tohoku J. Exp. Med..

[166] Gurbay A, Gonthier B, Signorini-Allibe N, Barret L, Favier A, Hincal F (2006). Ciprofloxacin-induced DNA damage in primary culture of rat astrocytes and protection by Vitamin E. Neurotoxicology.

[167] Simonin MA, Gegout-Pottie P, Minn A, Gillet P, Netter P, Terlain B (2000). Pefloxacin-induced achilles tendon toxicity in rodents: Biochemical changes in proteoglycan synthesis and oxidative damage to collagen. Antimicrob. Agents Chemother..

[168] Gurbay A, Hincal F (2004). Ciprofloxacin-induced glutathione redox status alterations in rat tissues. Drug Chem. Toxicol..

[169] Ouedraogo G, Morliere P, Santus R, Miranda, Castell JV (2000). Damage to mitochondria of cultured human skin fibroblasts photosensitized by fluoroquinolones. J. Photochem. Photobiol. B.

[170] Thuong-Guyot M, Domarle O, Pocidalo JJ, Hayem G (1994). Effects of fluoroquinolones on cultured articular chondrocytes flow cytometric analysis of free radical production. J. Pharmacol. Exp. Ther..

[171] Golomb BA, Devaraj S, Messner AK, Koslik HJ, Han JH, Yik B (2021). Lower blood malondialdehyde is associated with past pesticide exposure: Findings in Gulf War illness and healthy controls. Milit. Med. Res..

[172] Golomb BA, Koslik HJ, Christians U, Ritchie J, Wilson P, Elkins N, Klawitter J, Klawitter J, Smith D, Repine JE (2019). Depressed prostaglandins and leukotrienes in veterans with Gulf War illness. J. Environ. Sci. Health B.

[173] Jin X, Li F, Liu B, Zheng X, Li H, Ye F, Chen W, Li Q (2018). Different mitochondrial fragmentation after irradiation with X-rays and carbon ions in HeLa cells and its influence on cellular apoptosis. Biochem. Biophys. Res. Commun..

[174] Steele L, Lockridge O, Gerkovich MM, Cook MR, Sastre A (2015). Butyrylcholinesterase genotype and enzyme activity in relation to Gulf War illness: Preliminary evidence of gene-exposure interaction from a case-control study of 1991 Gulf War veterans. Environ. Health.

[175] Haley RW, Kramer G, Xiao J, Dever JA, Teiber JF (2022). Evaluation of a gene-environment interaction of PON1 and low-level nerve agent exposure with Gulf War illness: A prevalence case-control study drawn from the U.S. Military Health Survey's National Population Sample. Environ. Health Perspect..

[176] Fisher S, Bryant SG (1992). Postmarketing surveillance of adverse drug reactions: Patient self-monitoring. J. Am. Board Fam. Pract..

[177] Cham S, Evans MA, Denenberg JO, Golomb BA (2010). Statin-associated muscle-related adverse effects: A case series of 354 patients. Pharmacotherapy.

[178] Hoffman KB, Kraus C, Dimbil M, Golomb BA (2012). A survey of the FDA's AERS database regarding muscle and tendon adverse events linked to the statin drug class. PLoS ONE.

[179] Savarese DM, Savy G, Vahdat L, Wischmeyer PE, Corey B (2003). Prevention of chemotherapy and radiation toxicity with glutamine. Cancer Treat. Rev..

[180] Finkel T (2012). Relief with rapamycin: mTOR inhibition protects against radiation-induced mucositis. Cell Stem Cell.

[181] Iglesias-Bartolome R, Patel V, Cotrim A, Leelahavanichkul K, Molinolo AA, Mitchell JB, Gutkind JS (2012). mTOR inhibition prevents epithelial stem cell senescence and protects from radiation-induced mucositis. Cell Stem Cell.

[182] Talwar S, House R, Sundaramurthy S, Balasubramanian S, Yu H, Palanisamy V (2014). Inhibition of caspases protects mice from radiation-induced oral mucositis and abolishes the cleavage of RNA-binding protein HuR. J. Biol. Chem..

[183] Huang EY, Leung SW, Wang CJ, Chen HC, Sun LM, Fang FM, Yeh SA, Hsu HC, Hsiung CY (2000). Oral glutamine to alleviate radiation-induced oral mucositis: A pilot randomized trial. Int. J. Radiat. Oncol. Biol. Phys..

[184] Yavas C, Yavas G, Acar H, Toy H, Yuce D, Akyurek S, Ata O (2013). Amelioration of radiation-induced acute inflammation and mucosal atrophy by beta-hydroxy-beta-methylbutyrate, l-glutamine, and l-arginine: Results of an experimental study. Support Care Cancer.

[185] Vidal-Casariego A, Calleja-Fernandez A, Ballesteros-Pomar MD, Cano-Rodriguez I (2013). Efficacy of glutamine in the prevention of oral mucositis and acute radiation-induced esophagitis: A retrospective study. Nutr. Cancer.

[186] Chattopadhyay S, Saha A, Azam M, Mukherjee A, Sur PK (2014). Role of oral glutamine in alleviation and prevention of radiation-induced oral mucositis: A prospective randomized study. South Asian J. Cancer.

[187] Eda K, Uzer K, Murat T, Cenk U (2016). The effects of enteral glutamine on radiotherapy induced dermatitis in breast cancer. Clin. Nutr..

[188] Holler V, Buard V, Gaugler MH, Guipaud O, Baudelin C, Sache A, Perez Mdel R, Squiban C, Tamarat R, Milliat F (2009). Pravastatin limits radiation-induced vascular dysfunction in the skin. J. Investig. Dermatol..

[189] Wardman P, Folkes LK, Bentzen SM, Stratford MR, Hoskin PJ, Phillips H, Jackson S (2001). Influence of plasma glutathione levels on radiation mucositis. Int. J. Radiat. Oncol. Biol. Phys..

[190] Ma H, Zhang X, Bai M, Wang X (2007). Clinical effects of lianbai liquid in prevention and treatment of dermal injury caused by radiotherapy. J. Tradit. Chin. Med..

[191] Haddad MC, Khouzami RA, Saad HA, Azzi MC (2004). Imaging findings of radiation enteritis. J. Med. Liban.

[192] Cho S, Breedlove JJ, Gunning ST (2008). Radiation recall reaction induced by levofloxacin. J. Drugs Dermatol..

[193] Matthews RH, Ercal N (1996). Prevention of mucositis in irradiated head and neck cancer patients. J. Exp. Ther. Oncol..

[194] Yildiz, S. *et al.* Hyperbaric oxygen therapy used to treat radiation injury: Two case reports. *Ostomy Wound Manag.* **52**(5), 14–16, 18, 20 (2006). 16773750

[195] Tumerdem-Ulug B, Kuran I, Ozden BC, Mete O, Kemikler G, Aktas S, Calik B (2011). Does hyperbaric oxygen administration before or after irradiation decrease side effects of irradiation on implant sites?. Ann. Plast. Surg..

[196] Heuser G, Heuser SA (2017). Functional brain MRI in patients complaining of electrohypersensitivity after long term exposure to electromagnetic fields. Rev. Environ. Health.

[197] Johansson O (2015). xElectrohypersensitivity: A functional impairment due to an inaccessible environment. Rev. Environ. Health.

[198] Clegg, F. *Electrohypersensitivity is Real*. (The Huffington Post, 2013) (huffingtonpost.ca/frank-clegg/post_5393_b_3745157.html).

[199] Golomb BA, Koslik HJ, Han JH, Preger Guida AH, Hamilton G, Kelley RI (2021). A pilot study of bioenergetic marker relationships in Gulf War illness: Phosphocreatine recovery vs. citric acid cycle intermediates. Int. J. Environ. Res. Public Health.

[200] Naviaux RK, Naviaux JC, Li K, Wang L, Monk JM, Bright AT, Koslik HJ, Ritchie JB, Golomb BA (2019). Metabolic features of Gulf War illness. PLoS ONE.

[201] Fukuda K, Nisenbaum R, Stewart G, Thompson WW, Robin L, Washko RM, Noah DL, Barrett DH, Randall B, Herwaldt BL (1998). Chronic multisymptom illness affecting Air Force veterans of the Gulf War. J. Am. Med. Assoc..

[202] Institute of Medicine Committee to Assess the S, Efficacy of the Anthrax V. In: *The Anthrax Vaccine: Is It Safe? Does It Work?* (eds Joellenbeck, L. M. *et al.*) Copyright 2002 by the National Academy of Sciences. All rights reserved. (National Academies Press, 2002). 25057597

[203] Robbins ME, Zhao W (2004). Chronic oxidative stress and radiation-induced late normal tissue injury: A review. Int. J. Radiat. Biol..

[204] Meier EL, Mink van der Molen DR, Lansdorp CA, Batenburg MCT, van der Leij F, Verkooijen HM, Boonstra O, Hummelink S, Ulrich DJO (2023). Hyperbaric oxygen therapy for local late radiation toxicity in breast cancer patients: A systematic review. Breast.

[205] Morgenstern H, Greenland S (1990). Graphing ratio measures of effect. J. Clin. Epidemiol..

[206] Department of Defense: Research Advancement Award. Funding Opportunity Number:W81XWH-20-GWIRP-RAA. (Gulf War Illness Research Program, 2020).

[207] Committee on the Development of a Consensus Case Definition for Chronic Multisymptom Illness in - Gulf War V, Board on the Health of Select P, Institute of M. In *Chronic Multisymptom Illness in Gulf War Veterans: Case Definitions Reexamined.* edn. Copyright 2014 by the National Academy of Sciences. All rights reserved. (National Academies Press, 2014). 25590117

[208] Bui, L., Nguyen, E., Dinkeloo, E., Ritchie, J. & Golomb, B. A. Nuclear and Mitochondrial Genetics Together Determine Gulf War Illness Severity and Symptom Profile. *Gulf War Illness 2020 State of the Science Virtual Conference* 2020, (2020) https://va-eerc-ees.adobeconnect.com/_a1089657440/plixyd1089657440rh1089657444ht/?OWASP_CSRFTOKEN=1089657487e1827022744e1089657442d1089657447d1032270316e1089657441df1089657445a1089657442b1089657441c1089657442abb1089657425c1089651687a1089657466f1089657447eb1089657442ce1089657448b1089657449cba1089657444&proto=true.

[209] Rothman KJ (1990). No adjustments are needed for multiple comparisons. Epidemiology.

