# Supplementary material for: Susceptibility to radiation adverse effects in veterans with Gulf War illness and healthy civilians
Source: Sci Rep. 2024 Jan 9;14:874. doi: 10.1038/s41598-023-50083-7 (PMC10776672; doi:10.1038/s41598-023-50083-7)
Supplement: Supplementary file 1 — Supplementary Information. [file 41598_2023_50083_MOESM1_ESM.docx]

| **Supplement Table 1.** Radiation adverse effect propensity*: univariable predictors† | | | | | | |
| --- | --- | --- | --- | --- | --- | --- |
| Exposure | All | | Case | | Control | |
|  | Z | P | Z | P | Z | P |
| Carbon monoxide | 5.89 | 0.00 | 4.68 | 0.00 | 2.51 | 0.01 |
| Diesel fumes | 4.86 | 0.00 | 3.15 | 0.00 | 2.51 | 0.01 |
| Kerosene | 4.60 | 0.00 | 3.00 | 0.00 | 2.64 | 0.01 |
| Degreaser | 3.72 | 0.00 | 2.66 | 0.01 | 1.52 | 0.13 |
| Petroleum products | 3.30 | 0.00 | 1.03 | 0.31 | 3.38 | 0.00 |
| Naphtha | 3.27 | 0.00 | 2.30 | 0.02 | 1.59 | 0.11 |
| Carbamate pesticides | 3.96 | 0.00 | 2.30 | 0.02 | 2.68 | 0.01 |
| Moth repellent | 3.88 | 0.00 | 3.10 | 0.00 | 1.12 | 0.26 |
| Pesticides on clothes or bedding | 3.49 | 0.00 | 0.87 | 0.39 | 4.0 | 0.00 |
| Cobalt | 4.57 | 0.00 | 2.34 | 0.02 | 3.68 | 0.00 |
| Iron | 4.40 | 0.00 | 2.66 | 0.01 | 2.51 | 0.01 |
| Chromium | 4.19 | 0.00 | 2.59 | 0.01 | 2.89 | 0.00 |
| Arsenic | 3.61 | 0.00 | 2.13 | 0.03 | 2.64 | 0.01 |
| Mercury | 3.24 | 0.00 | 2.10 | 0.04 | 1.71 | 0.09 |
| Xray radiation | 4.16 | 0.00 | 3.18 | 0.00 | 1.84 | 0.07 |
| Other radiation | 3.83 | 0.00 | 2.85 | 0.00 | 0.32 | 0.75 |
| Radiation therapy | 2.60 | 0.01 | 3.22 | 0.00 | -0.51 | 0.61 |
| Radioactive chemicals | 4.10 | 0.00 | 3.31 | 0.00 | 0.88 | 0.38 |
| Cologne | 3.59 | 0.00 | 3.86 | 0.00 | 0.75 | 0.45 |
| Twinrx | 2.83 | 0.00 | 2.04 | 0.04 | 0.90 | 0.37 |
| Yellow fever | 3.05 | 0.00 | 2.25 | 0.02 | 0.83 | 0.40 |
| *Summed Exposures*‡ | *4.75* | *0.00* | *3.25* | *0.00* | *2.02* | *0.04* |
| * Radiation adverse effect propensity = summed radiation adverse effect score/summed radiation exposure score  † Nonparametric test of trend. From 133 exposure questions (drug, chemical, radiation).  ‡ Summing reported exposures  Carbon monoxide exposure as no, maybe, yes, were tied to the following RadAE propensity values, respectively  All: 0.021, 0.13, 0.22 (10x greater in carbon monoxide exposed than unexposed)  Control: 0.019, N/A, 0.19 (10x greater in carbon monoxide exposed than unexposed)  Case: 0.034, 0.20, 0.26 (~8x greater in carbon monoxide exposed than unexposed) | | | | | | |

| **Supplement Table 2.** Radiation adverse effect propensity*: effect of adding GWI severity or chemical sensitivity to the multivariable model† | | | | | | | | |
| --- | --- | --- | --- | --- | --- | --- | --- | --- |
| **Supplement Table 2A.** Adding GWI severity ‡ | | | | | | | | |
| Predictors | All adjusted for GWI case status  N=81 | | | GWI cases  N=41 | | | | |
|  | OR (SE) | 95% CI | P | OR (SE) | 95% CI | | P | |
| Carbon monoxide | 33.1  (25.9) | 7.16, 153 | <0.001 | 115  (115) | 16.1, 814 | | <0.001 | |
| Twinrx | 3.67  (3.18) | 0.673 20.0 | 0.13 | 4.16  (5.75) | 0.277, 62.5 | | 0.30 | |
| Diesel fume | 9.71  (8.71) | 1.67, 56.3 | 0.011 | 11.7  (12.7) | 1.38, 99.0 | | 0.024 | |
| GWI severity ‡ | 1.06  (0.0284) | 1.01, 1.12 | 0.025 | 1.08  (0.0403) | 1.00, 1.16 | | 0.036 | |
| Case | 0.0312  (0.0391) | 0.00269, 0.362 | 0.006 |  | | | | |
| *Model Performance* | PseudoR^2^ = 0.30, Model P < 0.0001 | | | PseudoR^2^ = 0.36, Model P < 0.0001 | | | | |
| **Supplement Table 2B.** Adding chemical sensitivity § | | | | | | | | |
| Predictors | All adjusted for GWI case status  N=81 | | | GWI Cases  N=41 | | | | |
|  | OR (SE) | 95% CI | P | OR (SE) | | 95% CI | | P |
| Carbon monoxide | 31.4 (25.7) | 6.31, 156 | <0.001 | 108 (136) | | 8.99, 1290 | | <0.001 |
| Twinrx | 4.83 (2.93) | 1.47, 15.9 | 0.010 | 5.36 (3.94) | | 1.27, 22.6 | | 0.022 |
| Diesel fume | 12.4 (12.6) | 1.71, 90.6 | 0.013 | 28.7 (69.3) | | 0.255, 3239 | | 0.16 |
| Chemical sensitivity § | 4.21 (2.32) | 1.43, 12.4 | 0.009 | 7.53 (6.09) | | 1.54, 36.7 | | 0.013 |
| Case | 0.141 (0.126) | 0.0246, 0.813 | 0.028 |  | | | | |
| *Model Performance* | Pseudo R^2^ = 0.29, Model P = 0.0001 | | | PseudoR^2^ = 0.35, Model P < 0.0001 | | | | |
| * Radiation adverse effect propensity = summed radiation adverse effect score/summed radiation exposure score  † Ordinal logit with robust standard errors. Combined GWI cases and controls.  ‡ GWI severity gauged by GWI Kansas criteria symptom score (summed ratings across Kansas criteria symptom queries).  § Chemical sensitivity variable, binary (0 = absent, 1 = present)  GWI = Gulf War illness. OR = odds ratio. SE = standard error. CI = confidence interval. P = probability.  If both variables (chemical sensitivity and GWI severity) are added (not shown), each becomes borderline significant due to their collinearity (p ~0.060 for each, in the full sample). Carbon monoxide remains strongly significant (p<0.001, both in the full sample, and in cases separately), diesel-fuel is restored to significance in all and in cases (0.008 and 0.038 respectively), and case status remains a significant negative predictor of RadAE propensity in the full sample (p=0.003). Twinrx drops out as a predictor with adjustment for GWI severity, with or without added adjustment for chemical sensitivity. | | | | | | | | |

**Supplement 3**. Exposures assessed.

Some exposures were not cited by any participants. For exposures queried in the Gulf-specific and also the general exposure surveys, a Gulf War veteran was characterized as having the exposure if it was designated in either the general or Gulf-specific exposure survey.

**Supplement 3a**. General survey exposure: exposures assessed

Dry cleaned clothing

Fabric softener, dryer sheets

Chemical cleaners

Burning fuels

Carbon monoxide

Diesel or petrochemical fumes

Kerosene

Diesel or petrochemical fuel on skin

Petroleum products

Degreasing solutions

Other solvents, thinning agents

Acetone

Jet fuel

Fuel storage

Solvents

Paint, paint strippers

Napthas

Black flag

DEET

Dursban

Flea or tick medicine

Head lice treatment

Organochlorines

Organophosphate – insecticides

Fly spray

Moth repellent

Insect repellent

Carbamate pesticides

Pesticide cream or spray on skin

Pesticide on clothes/bedding

Pyrethroid pesticides/pest repellants

Saw in the area in which you lived or fogged or sprayed with pesticides

Other pesticides/insects

Raid

Regular pesticide treatment at work

Wood treatment

Roundup

Regular herbicide treatment or work

Other herbicides

Selenium

Cadmium

Copper

Iron

Arsenic

Chromium

Cobalt

Lead

Manganese

Mercury

Zinc

Thallium

Radiation therapy for cancer or other conditions

Other radiation

X-ray radiation

Radioactive chemicals

Aerosol sprays

Air freshener

Animal spray

Asbestos

Automotive products

Personal care products

Cologne/scented products

Drinking diet soda

Drywall dust

Office exposures

Make-up use

Use of suntan lotion or sunscreen

Water treatment chemicals

Antimalarial drugs

Ciprofloxacin or other fluoroquinolones

Chemotherapy

Doxycycline

Botox

Anthrax vaccine

Botulinum toxoid vaccine

Cholera vaccine

Hepatitis A vaccine

Hepatitis B vaccine

TwinRx Hep A and B vaccine

Immune globulin vaccine

Meningococcal vaccine

MMR – measles mumps rubella vaccine

Pertussis vaccine

Plague vaccine

Polio vaccine

Tetanus vaccine

Typhoid vaccine

Yellow fever vaccine

**Supplement 3b**. Gulf-specific exposures assessed.

NBC suits

Chemical or nerve gas attack

Heard chemical alarm sound

Gas mask use

Respraying vehicles

Chemical agent resistant compound paint

Other paints or solvents

Diesel or petrochemical fuel on skin

Diesel or petrochemical fumes

Exhaust from heaters or generator

Munition fumes

Inhaled smoke from oil-well fires

Saw smoke (from oil well fires)

Burned jet fuel

Burning rubbish/burn pits

Flea collars

Insect repellent

Saw the area in which a in which you lived sprayed with pesticides

Pesticide sprayed in quarters

Pesticide treatment of uniform

Permethrin treated uniform

Pesticides on clothing or bedding

Pesticide handling

Personal pesticide

Mustard gas

Ate local food

Got food poisoning

Unit got food poisoning

Drank diet soda

Drank water from desert bag

Drank contaminated water

Bathed in local pond/water

Water treatment chemicals

Antimalarials

Ciprofloxacin

Doxycycline

Pyridostigmine bromide pills

Less than 4 hours of sleep in 24 hour period

Tented accommodations

Temporary wooden structure

Sandstorms

Sunscreen

Arm immunization

Butt immunization

Anthrax vaccine

Botulinum toxoid vaccine

Cholera vaccine

Hepatitis A + B vaccine

Immune globulin

Meningococcal vaccine

Pertussis vaccine

Plague vaccine

Polio vaccine

Tetanus vaccine

Typhoid vaccine

Yellow fever vaccine

Contact with POWs

Involved in air combat

Involved in ground combat

Combat-related injury

Direct contract destroyed enemy vehicle (so, DU)

Saw destroyed enemy vehicle

Direct contact vehicle exposed to friendly fire

Saw dismembered bodies

Saw Americans killed

Saw Iraqis killed

Direct contact with dead animals

Saw dead animals

Witnessed someone dying

Came under small arms fire

Danger/direct combat

Scud missile exploded within one mile

Medical attention
